# Image key information processing using convolutional neural network and rotational invariant-hierarchical max pooling algorithm

**DOI:** 10.1371/journal.pone.0324504

**Published:** 2025-05-27

**Authors:** Guangmei Ma

**Affiliations:** School of Literature, Xi’an Siyuan University, Xi’an, China; Sri Krishna College of Engineering and Technology, INDIA

## Abstract

In the information age, the effectiveness of image processing determines the quality of a large number of image analysis tasks. A fusion algorithm-based processing technique was proposed to process key image information. A feature dictionary was introduced as the matching template model and the standard model. The convolutional layer sampling feature block optimization was carried out using image segmentation ideas. The optimal threshold of the image to be segmented was obtained using the least squares method. The feature extraction layer was structurally supplemented and expressed at multiple scales in a two-dimensional linear graph. In the method training loss test, the research method achieved a loss value that dropped to near 0 after 32 iterations when training in low-contrast images. When testing the processing time of image key information, the research method achieved a processing time of 183ms when the image contained 6 features. When conducting scale ratio change testing, the research method achieved the highest image processing accuracy at a scale ratio of 1.0, which was 95.7%. This indicated that the research method had higher accuracy in processing key image information and higher efficiency. This research method can provide certain technical support for image recognition and feature extraction.

## 1 Introduction

The advent of electronic information technology has led to a marked increase in the size and complexity of image data, which has emerged as a critical component of electronic information data. Extraction and processing of key information in image processing determine the quality of image processing. Image processing technology is becoming an important branch of computer vision. As the field of big data and artificial intelligence continues to evolve, research in the realm of image key information processing is undergoing a significant surge of interest from both academic circles and industry leaders [1,2]. The commonly used image key information processing technologies currently include Convolutional Neural Network (CNN), transfer learning, digital image processing techniques, etc. CNN can automatically extract hierarchical features from images by simulating the mechanisms of the human visual system. The multi-layer structure of CNN can capture multi-level abstract features from simple edges to complex object parts [3]. However, CNN has high requirements for computing resources when used alone, which leads to limitations in many scenarios. Transfer learning can improve training efficiency in image recognition by transferring knowledge learned in one domain to another domain to aid in the learning of target tasks [4]. Nevertheless, the efficacy of transfer learning is constrained by the degree of similarity between the source task and the target task. If there is a significant difference between the two, transfer learning may not achieve the expected results. Digital image processing technology has poor real-time processing performance when facing complex images [5]. Hierarchical Max Pooling (HMAX) is a model that simulates the processing mechanisms of the human visual cortex. HMAX extracts key features of images through a hierarchical max pooling operation. In this context, this study attempts to innovatively combine CNN and HMAX and propose a new comprehensive image key information processing technology. Feature dictionaries and rotation invariant optimization are innovatively utilized to obtain the Rotational Invariant-Hierarchical Max Pooling (RI-HMAX) algorithm. The objective of this study is to furnish specific technical references for the domain of image processing through technological innovation.

The contribution of the research lies in: (1) The method presents an innovative approach to image critical information processing and combines the efficient feature extraction capability of CNN with rotational invariance and hierarchical maximum pooling, which achieves highly robust processing of multi-angle and multi-scale images. (2) The utilization of Harris corner detection and feature normalization has been demonstrated to address the limitations of CNNs in expressing features in rotating scenes. The multi-scale feature representation, based on pixel rings, has been demonstrated to effectively enhance the model’s rotation and scaling adaptability, thereby providing technical support for complex image scene processing. (3) This study designs a template matching similarity metric based on Euclidean distance combined with rotational normalization of feature points to significantly improve the accuracy of feature matching.

This study is mainly conducted from four sections. First, the research achievements related to HMAX and image processing are discussed. Second, an image processing and recognition method combining CNN and RI-HMAX is designed. Then the effectiveness of the research design method is analyzed. Finally, a summary is provided for the entire article.

## 2 Related works

Image data are increasingly being applied in various industries. A considerable number of scholars have begun to direct their attention toward the significance of image key information processing technology. A number of scholars have conducted research on image processing technology. X Zhou et al. proposed a simplified target imaging method for the conversion problem of radar images. During the process, image interpolation and coordinate transformation techniques were introduced, and a distance algorithm was used to construct a near-field correction model. This proposed method had high robustness and effectively completed image conversion [6]. Scholar Xu applied machine learning to extract longitudinal phase information from images. Traditional methods had problems such as slow extraction speed and susceptibility to getting stuck in local optima. Compared to classical methods, machine learning methods still maintained better output quality images even when data noise was high [7]. M Kosowski et al. proposed a conversion method that integrated time and voltage parameters for Complementary Metal Oxide Semiconductor (CMOS) image conversion. CMOS was a semiconductor technology used in the fabrication of electronic circuits, including image sensors in cameras. It converted light into electronic signals using photodiodes and amplifiers for each pixel, enabling efficient and fast imaging. During the process, the image pixels of the light-emitting diode were extracted, and the reference voltage was regulated during the operation of the single slope analog-to-digital converter. The experimental results showed that the proposed method achieved an average image conversion speed of 289ms per image in low pixel areas [8]. Sichkar V N and Kolyubin S A proposed a detection model using the You Only Look Once (YOLO) algorithm for the detection and classification of traffic sign images. The YOLO algorithm was a real-time object detection algorithm designed to identify and classify objects in images. A distinctive feature of YOLO is its ability to process an entire image in a single pass, a capability that facilitates faster processing times compared to other algorithms. It employed a single neural network to predict bounding boxes and class probabilities concurrently. During the process, the shape was used as a reference for symbol classification, and Red-Green-Blue images were used for model training. The experimental results demonstrated that the proposed method exhibited a high detection accuracy, with an average of 96.3%, and was feasible in practical application [9]. Kikuta H et al. proposed a method combining optical system projection to address the high visibility image processing. During the process, the fuzzy features were quantified, and a filter was used to correct the input image data. This process extracted the impact of the imaging optical path on the image. The experimental results demonstrated that the proposed method could effectively enhance the accuracy of image processing, with an improvement of over 5% compared to the baseline algorithm [10].

Some scholars have conducted relevant research on HMAX. Akbarpour M et al. proposed a method based on HMAX to address the object detection. During the process, a training sample pool was extracted from the objects, and unsupervised object detection was used to optimize the sample data. Patches were sorted to improve information discrimination. The experimental results demonstrated that the proposed method exhibited both adequate operational sensitivity and computational speed, with an average single run time maintained within 250 ms [11]. Karpat E et al. proposed an HMAX-based method for optimizing antenna parameters. During the process, a rectangular patch antenna was designed. Meanwhile, the resonant frequency was extracted using a design variable function to minimize the difference between the calculated frequency and the target frequency. The experimental results showed that the proposed method had good dimensional accuracy, with an average of 97.2%, and could maintain high computational speed [12]. Pawani K et al. proposed a method combining HMAX for solving the scheduling problem of cogeneration. During the process, wavelet mutation was used to circumvent local minima of individuals. The mucus behavior and reproductive stage were used as the basis for the algorithm, which was developed using multi-source experiential learning to adapt to dimensional complexity.The experimental results showed that the proposed method had high solution quality and convergence speed, and could be trained in less than 100 iterations [13]. Sun H et al. proposed a technique combining HMAX for solving examination problems in urological surgery. During the process, ultrasound examination was used to obtain detection source data. Meanwhile, ultrasound guided obturator nerve block combined with adaptive algorithm was used for data analysis. The experimental results demonstrated that the solution time of the proposed method could be maintained within 500 ms, which could effectively improve the quality of ultrasound-guided surgery [14]. Shaheen A M et al. proposed a method based on HMAX for the economic scheduling problem of cogeneration. Four testing systems were established during the process. Concurrently, the solution scale of the systems underwent differentiation to balance the supply of heat, dispatch power generation, and fuel costs. Corresponding business restrictions were also incorporated. The experimental results showed that the proposed method had good solving efficiency, with a single solving time kept within 300ms [15].

Some scholars have studied the application methods of computer technology. Madebo M et al. proposed an intelligent model reference adaptive control method based on neural networks for robust tracking control of quadcopter drones under external disturbances and parameter changes. A dynamic system was constructed using a Newtonian quaternion model, feedforward and recurrent neural networks were trained offline to initialize controller parameters, and online learning algorithms were developed to update the network in real time. The simulation results demonstrated that this method exhibited superior tracking performance, disturbance rejection capability, and reduced control cost in comparison to traditional adaptive control, thereby substantiating the viability of its real-time implementation [16]. Ayalew et al. proposed a comprehensive algorithm combining adaptive Monte Carlo localization for path planning of mobile service robots in unknown dynamic environments. The generation of global paths was achieved through the implementation of bi-directional fast exploration of random trees, while local planning was executed by dynamic windowing methods. The recognition of objects was facilitated by image algorithms. Simulation and experimental results showed that this method outperformed existing algorithms and was suitable for dynamic environments [17]. Ayalew et al. proposed a hybrid algorithm combining adaptive Monte Carlo localization for intelligent mobile robot path planning in unknown dynamic environments. The localization was achieved through adaptive Monte Carlo positioning, which solved the problem of path planning in dynamic environments [18].

In summary, although HMAX has been studied and applied in many industries, research on image processing is still relatively scarce. A small amount of research also has certain limitations. Therefore, this study attempts to use HMAX for image processing technology design and seeks optimization approaches to design a high-performance image processing technology.

## 3 Image processing and recognition method integrating CNN and RI-HMAX algorithms

This section provides a detailed explanation of the techniques used and construction of image key information processing techniques. The basic construction of image processing technology is studied through HMAX. The regionprops function is combined to measure image region attributes and optimize the feature extraction process. Multi-scale representation is performed in the two-dimensional linear graph, extracting the content of the image and corresponding key data.

### 3.1 Image processing technology based on improved HMAX

**Fig 1:**
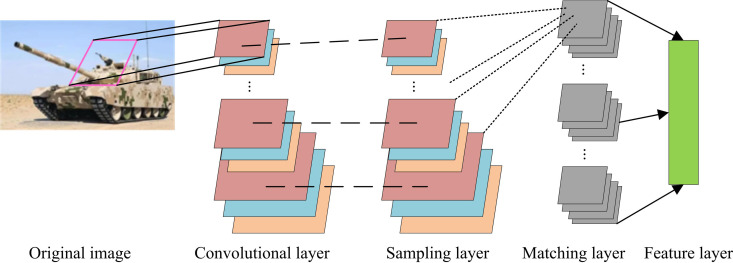
Standard model selected for research.

The advent of computer technology has led to a marked increase in the complexity of image processing. The ability to extract key information from images has emerged as a critical facet of this process. HMAX is a biologically inspired feature analysis algorithm that mimics the working principles of vision and is capable of extracting key information from images. Image processing and recognition methods are constructed based on HMAX. This study uses HMAX, which introduces feature dictionaries as matching templates and the standard model in Fig 1.

In Fig 1, the standard HMAX selected consists of convolution, sampling, matching, and feature layers. The model’s input is usually a grayscale image. The output is a set of feature vectors for the test image, which can be passed into various classifiers for training classification and identifying image categories. In the Convolutional Layer (CL), Gabor filters are used to filter and calculate the input image, resulting in multiple sets of response maps. Gabor filtering is represented by equation (1).


$$\mathrm{\backslash [}\left\{ \begin{array}{c} @lF\left(x,y\right)=\exp\left(-\frac{(x\cos\theta +y\sin\theta {)}^{2}+\gamma^{2}(-x\sin\theta +y\cos\theta {)}^{2}}{2\sigma^{2}}\right)\\ \times \cos\left(\frac{2\pi (x\cos\theta +y\sin\theta )}{\lambda }\right) \end{array}\right.\mathrm{\backslash ]}$$ (1)

In equation (1), F(x,y) represents the filtering calculation result. γ represents aspect ratio. λ represents wavelength. σ represents effective bandwidth. θ represents direction. (x,y) represents the position of the filtering window. Adjacent response maps are divided into a frequency band according to different scales. The maximum value of the response map in the frequency band is aggregated in the sampling layer, thereby converting the response map to the maximum value of the downsampling process. The corresponding maximum values of two adjacent scales within the same frequency band are recalculated. When processing key information in images, it is necessary to randomly extract different class features and aggregate them into one training result [19,20]. The output of the sampling layer and the extraction of template feature blocks are matched and calculated, represented by equation (2).


$$Y=\exp\left(-\gamma {\left\|X-P_{i}\right\|}^{2}\right)$$ (2)

In equation (2), Y represents the matching calculation result. X represents the output of the sampling layer. $P_{i}$ represents extracting template feature blocks. The matching layer traverses the responses of all groups, searches for the maximum value, and obtains an N-dimensional vector. To enhance the reproducibility of the algorithm and mitigate computational complexity, research is being conducted on the optimization of CL sampling feature blocks through the application of image segmentation methods. The feature extraction content of Gabor is refined through local contrast significance detection. The saliency value of pixels in the image is represented by equation (3).


$$SalS\left(I_{K}\right)=\sum_{\forall I_{i}\in I}\left\|I_{K}-I_{i}\right\|$$ (3)

In equation (3), $SalS\left(I_{K}\right)$ represents the saliency value of the K th pixel in the I th image. $I_{K}$ represents the K th pixel’s position in the I th image. $I_{i}$ represents the average position of all pixels. Pixel frame number is introduced, and the color measurement method is set to Red Green Blue color combination to generate saliency detection images. The maximum inter-class variance method is used for clustering segmentation, and important information is separated from the background by image processing. The optimal threshold of the image to be segmented is obtained using the least squares method. The grayscale histogram of the segmented image is used for inter-class differentiation to separate parts with different grayscales [21,22]. The least squares method provides a robust mathematical framework for processing image data by minimizing the sum of squared errors to find the best-fitting parameters. This method is more accurate than the Otsu method in the case of image matching, which is required in research. Second, the Otsu method is generally effective in automatically determining thresholds. However, its performance is constrained by the contrast between the foreground and background of the image. In scenarios where contrast is minimal or the quality of the image is suboptimal, the Otsu method may not yield optimal segmentation outcomes. The threshold is searched for based on grayscale characteristics for image segmentation, resulting in foreground and background. The global mean of the image is represented by equation (4).


P1×m1+P2×m2=mG (4)

In equation (4), P1 represents the probability that pixels are classified as the first class. P2 represents the probability that pixels are classified as the second class. m1 represents the mean of the first type of image. m2 represents the mean of the second type of image. mG represents the global mean of the image. The inter class variance is represented by equation (5).


$$\sigma^{2}=P1P2{\left(m1-m2\right)}^{2}$$ (5)

**Fig 2:**
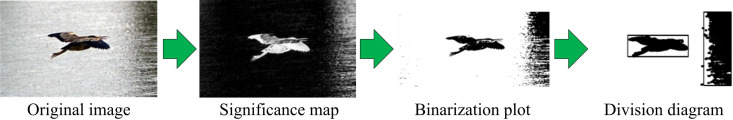
Significant region segmentation.

In equation (5), $\sigma^{2}$ represents the inter class variance. Fig 2 shows the segmentation of salient regions.

**Fig 3:**
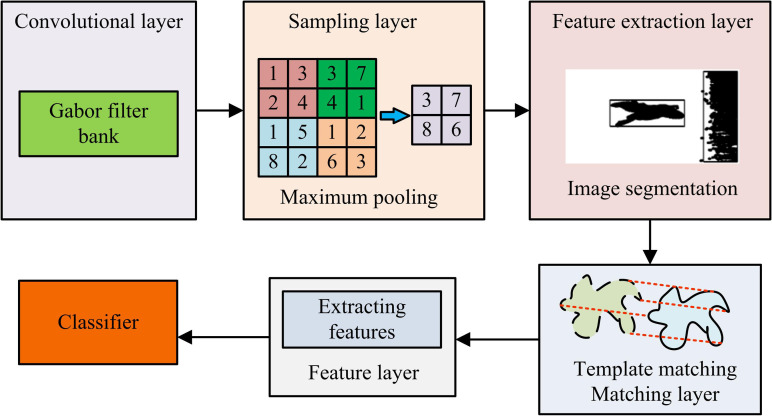
Improved HMAX model running process.

In Fig 2, the original image is subjected to local contrast saliency detection to obtain a saliency map, which is then binarized using the maximum inter class variance method. Based on the binarization results, the region props function is used to measure the image region attributes and mark the regions of interest. Regions of interest in binary images are extracted and labeled with rectangular boxes, resulting in significant region segmentation results labeled with matrix boxes [23,24]. During this process, the grayscale values are traversed to obtain the maximum inter-class variance, and the corresponding grayscale values are then used as thresholds as input parameters for the binarization function. Corrosion and expansion processes are inserted before binarization. Expansion is similar to domain expansion, where images are traversed at the center of the structural elements. The maximum value of all pixels within the coverage area is used to replace the current alignment element, connecting the small breaks of the foreground object. Corrosion bears a resemblance to the process of erosion, wherein the minimum value within the specified coverage area is replaced, the thin lines in the background are removed, and a segmentation area with enhanced connectivity is obtained. Fig 3 shows the improved HMAX operating process designed.

In Fig 3, the improved HMAX designed consists of a template learning stage consisting of convolutional and sampling layers during image processing. During template learning, the Gabor filter banks in CL are used for operation, and the sampling layer performs max pooling. During actual operation, the entire model structure works. Image segmentation sampling feature blocks are executed in the feature extraction layer, template matching is performed in the matching layer, and features are extracted in the feature layer. The features extracted by the feature layer are passed to the classifier for synthesis, which completes image processing.

### 3.2 Optimization of RI-HMAX image processing technology using CNN feature extraction layers

In actual image processing scenarios, there may also be situations of image rotation and scale changes. The performance of using improved HAMX is affected when processing images with changes in angle and scale [25,26]. To improve the processing performance of research methods, RI-HMAX is proposed based on HMAX. In RI-HMAX, the feature extraction layer mainly extracts key information of the image through Harris corner detection. The Harris corner algorithm value is represented by equation (6).


$$E_{x,y}=\sum_{u,v}w_{u,v}{\left|I\left(u+x,v+y\right)-I\left(u,v\right)\right|}^{2}$$ (6)

In equation (6), $E_{x,y}$ represents the corner value. $w_{u,v}$ represents the window function. I(u+x,v+y) represents the image being processed. I(u,v) represents the standard corner image. The window function is represented by equation (7).


$$w_{u,v}=\exp\left(-\frac{u^{2}+v^{2}}{2\delta^{2}}\right)$$ (7)

In equation (7), u represents the coordinates of the standard corner point on the x-axis. v represents the coordinates of the standard corner point on the y-axis. δ represents the window function coefficient. After Taylor expansion and retrieval, corner values are transformed into matrix expressions, and line and corner search is performed using parameter indicators, represented by equation (8).


$$P=\det M-k{\left(trace\left(M\right)\right)}^{T}$$ (8)

**Fig 4:**
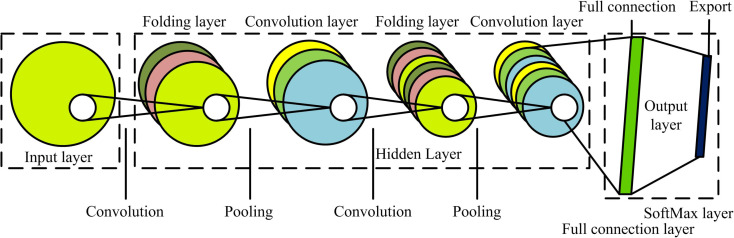
CNN structure.

In equation (8), P represents the parameter indicator value. M represents matrix expression. If the absolute value of P is small, then the area around the pixel is a plane. If P is a frame rate with a larger value, it is determined that the pixel is a corner point. If P is a large negative number, it is determined that the pixel is the content of the line. The corners detected in the image are considered as the center points of the extracted patches, and Harris corners are searched in the output data of the sampling layer [27,28]. This study proposes the incorporation of CNN into the feature extraction layer to enhance the accuracy of feature extraction and expand the applicability of the research method. The structure of CNN includes convolution, pooling, and fully connected layers in Fig 4.

In Fig 4, CNN can be divided into input, hidden, and output layers. The input layer directly receives data input. Convolutional and pooling layers both are attributable to hidden layers. CL has multiple convolutional units. Features extraction relies on convolution kernels [29,30]. The pooling layer can reduce the feature vectors output by CL, alleviate overfitting, and use overlapping and maximum pooling methods for data processing. The output layer contains CL and SoftMax. The fully connected layer contains multiple neuron nodes and performs data analyzing. CL’s feature extracting results are represented by equation (9).


$$r_{a}^{l}=f\left(H_{a}^{l}⊗g_{a}^{l}+b_{a}^{l}\right)$$ (9)

In equation (9), $r_{a}^{l}$ represents the a th neuron’s output value in the l th layer. $H_{a}^{l}$ represents the neuron’s convolutional kernel parameter. $g_{a}^{l}$ represents CL’s input value. $b_{a}^{l}$ represents a bias coefficient. ⊗ represents convolution operation. To reduce the running load, Winograd is used for convolution operation, represented by equation (10).


μ(F(m,r))=m+r−1 (10)

In equation (10), μ() stands for the decrease in computational complexity. F(m,r) stands for one-dimensional convolution with reduced complexity. m means the outputting size. r stands for filter’s size. A set of F(m,r) are added and overlapped to increase the dimensionality to two-dimensional, represented by equation (11).


μ(F(m×n,r×s))=(m+r−1\rightleft(n+s−1) (11)

In equation (11), F(m×n,r×s) stands for the two-dimensional convolution obtained by overlapping. n and s stand for another set of related sizes for one-dimensional convolutions. For a two-dimensional Winograd algorithm having the same size on rows and columns, this algorithm is performed again in the column, represented by equation (12).


$$\mathrm{\backslash [}Y=A^{T}\left[\left(GgG^{T}\right)⊙\left(B^{T}dB\right)\right]A\mathrm{\backslash ]}$$ (12)

**Fig 5:**
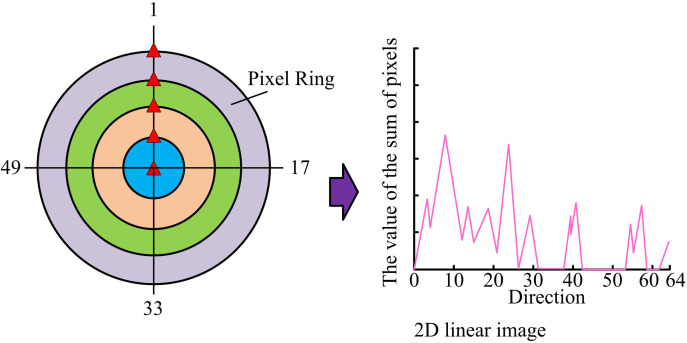
Pixel ring feature expression.

In equation (12), $A^{T}$ and $B^{T}$ stand for post-processing and input transforming matrix. G means a coefficient transforming matrix. g is an r×r coefficient matrix. d means an inputting matrix . These features are extracted synchronously using CNN and integrated with the results of Harris corner detection to obtain comprehensive feature extraction data of image key information. When performing feature representation, if the image is rotated or scaled, the gradient direction of pixels will change. At this point, it is not possible to directly use gradient direction histograms as feature descriptors. The pixels extracted from the original patch are rearranged and transformed into multiple pixel rings with different radii in Fig 5.

**Fig 6:**
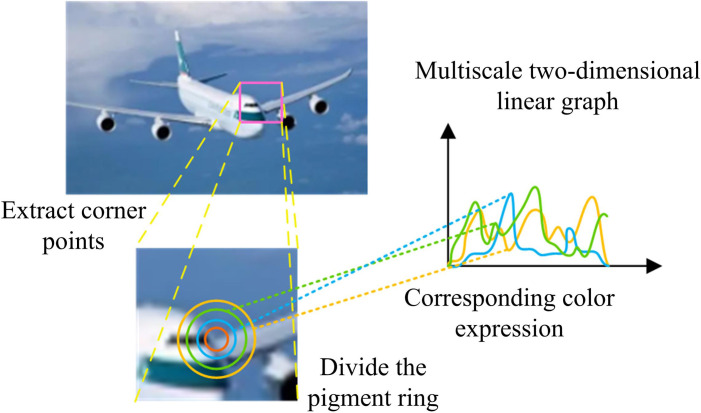
Multi-scale representation of two-dimensional linear graphs.

In Fig 5, when feature representation is performed by multiple pixel rings with different radii, all pixels with the same distance from the feature block are classified into one ring. Pixels on a ring of one radius are selected for conversion to a two-dimensional linear image. 64 coordinate values are set on the horizontal axis of a two-dimensional linear image, representing the direction of the pixel gradient. The vertical axis represents the accumulation of pixel values in that direction, and the origin is the center of the feature block. In a linear display, the predominant direction of feature blocks is frequently paramount, as the linear arrangement of pixels near corners often aligns with the edges or contours of the image. Normalizing the linear graph to the dominant direction reduces the feature changes caused by rotation, thereby improving the accuracy and robustness of template matching. The normalization process first identifies key points in the image. Corner points retain feature invariance after rotation. A subsequent analysis of the vertices is then conducted to ascertain the dominant direction within the image, which is identified as the direction exhibiting the most substantial feature distribution. After determining the dominant direction, the model rearranges the image features based on the obtained direction, so that the features are uniformly represented in the new direction. Multi-scale representation is performed in the two-dimensional linear graph in Fig 6.

In Fig 6, pixel features are extracted in three different radius loops in a two-dimensional linear graph, with each image overlapping with another pixel. When performing template matching, the Euclidean distance between pixels and the template to be matched is calculated. Meanwhile, a highly matching linear graph is obtained, which achieves scale and rotation invariance of the image. The rotation invariance module determines the principal direction of image key points through Harris corner detection, normalizes feature blocks, and rotates feature points to a unified principal direction to reduce feature bias caused by rotation. The extracted feature points are then rearranged into circular structures of varying radii by pixel rings. These structures are then converted into two-dimensional linear graph representations. This process ensures rotation invariance of the extracted features at different scales while preserving key details.To reduce the computational complexity in identifying key information, the patch-patch strategy is used. During testing, each detected corner feature block is compared with the feature blocks obtained during training, and similarity is calculated using Euclidean distance and Gaussian kernel projection. Euclidean distance is calculated as shown in equation (13).


$$d_{o}=\sqrt{\sum_{i=1}^{n}{\left(x_{i}-y_{i}\right)}^{2}}$$ (13)

**Fig 7:**
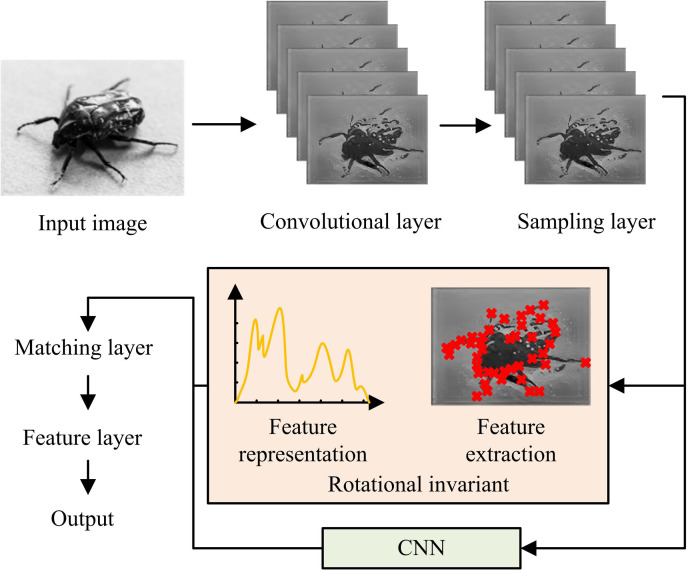
RI-HMAX image processing technology.

In equation (13), $d_{o}$ represents Euclidean distance. $x_{i}$ represents the i eigenvalue of the template pixel. $y_{i}$ represents the i th eigenvalue of the pixel to be matched. Fig 7 shows the constructed RI-HMAX image processing technology.

In Fig 7, when the constructed RI-HMAX image processing technology performs image key information processing, the rotation invariant module runs between the sampling layer and the matching layer. After entering CL, the original image enters the sampling layer. The results of the sampling layer are divided into two parts, one of which enters the feature extraction layer and the other enters the CNN. The feature extraction layer takes the detected image corners as the center points of the feature blocks. After feature representation and normalization, these data are combined with the feature data output by CNN and imported into the matching layer. The feature layer completes the synthesis and output of key image information.

## 4. Effectiveness analysis of optimized RI-HMAX image key information processing technology

The actual effectiveness of image key information processing technology was analyzed through performance testing and application analysis. Performance testing was conducted using training loss, feature recognition accuracy, and image key information processing calculation time. In practical applications, corner extraction, physical image segmentation, rotation invariant representation analysis, and scale ratio change test were conducted to determine the application effect.

### 4.1 Performance testing of optimized RI-HMAX image key information processing technology

The performance of the designed image key information processing technology that integrates CNN and RI-HMAX was tested. Speed Up Robust Features (SURF) and Orthogonal Matching Pursuit (OMP) algorithms were selected for comparison. The research method was abbreviated as CNN-RI-HMAX. The Graz dataset and Caltech101 dataset were selected for testing. Table 1 shows the basic software and hardware environment settings.

**Table 1:** The experimental basic environmental parameters.

Parameter variables	Parameter selection
CPU	Intel⑧CoreTM i7-6700HQ CPU
Memory	16G
GPU	NVIDIA GeForce GTX1660
Framework	Tensorflow
Operating system	Wndows10
Operating environment	PyCharm
Script	Python

**Fig 8:**
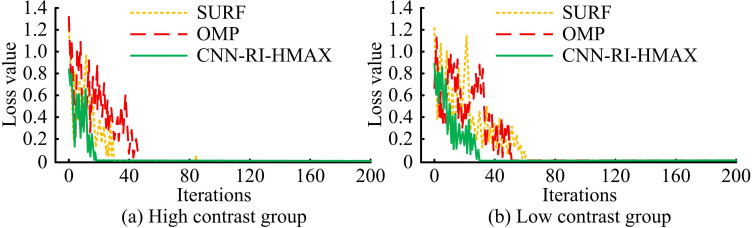
Method training loss testing.

Before conducting the test, the images were uniformly converted into grayscale images and the size was reduced to 300 × 200. The Graz dataset contained 1000 images, while the Caltech 101 dataset contained 9146 images. The dataset was divided into 80% training data and 20% testing data. The training loss results come from the training data, while the remaining testing results come from the testing data. The Michelson contrast index was employed to quantitatively analyze the image contrast. The contrast index value was classified as either a high contrast group or a low contrast group, depending on whether it exceeded or fell below the preset threshold of 0.6, respectively. The training loss of the method was analyzed separately in the high contrast group and the low contrast group, as shown in Fig 8.

In Fig 8, different methods had certain differences in the iterations required to achieve stability during training. In Fig 8(a), when SURF was trained in high-contrast images, the initial loss value reached 1.24. When the iteration reached 47 times, the loss value dropped to a position close to 0 and remained basically stable. When OMP was trained in high-contrast images, the initial loss value reached 1.32. When the iteration reached 31 times, the loss value dropped to a position close to 0 and remained basically stable. When training CNN-RI-HMAX in high-contrast images, the initial loss value was 0.83. When the iteration reached 19 times, the loss value dropped to a position close to 0 and remained basically stable. In Fig 8(b), when SURF was trained in low-contrast images, the initial loss value reached 1.22. When the iteration reached 62 times, the loss value dropped to a position close to 0 and remained basically stable. When OMP was trained in low-contrast images, the initial loss value reached 0.68. When the iteration reached 51 times, the loss value dropped to a position close to 0 and remained basically stable. When training CNN-RI-HMAX in low-contrast images, the initial loss value was 0.90. When the iteration reached 32 times, the loss value dropped to a position close to 0 and remained basically stable. This indicated that this research method had a faster training efficiency and a more stable training process.

**Fig 9:**
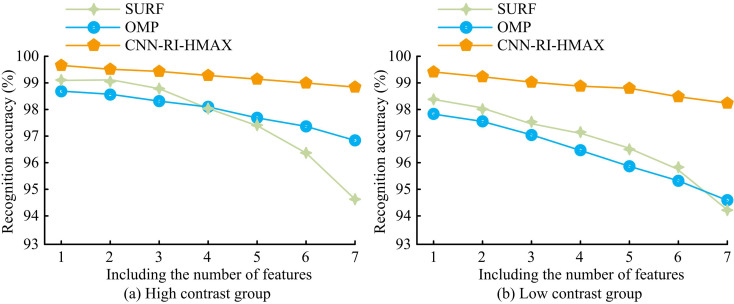
Feature recognition accuracy testing.

In Fig 9, the accuracy of image feature recognition for different methods decreased as the features in the image increased. In Fig 9(a), in high-contrast images, the feature recognition accuracy of SURF was 99.1% when the image contained one feature. The feature recognition accuracy decreased to 94.6% when the image contained 7 features. The feature recognition accuracy of OMP was 98.7% when the image contained one feature. The feature recognition accuracy decreased to 96.8% when the image contained 7 features. The feature recognition accuracy of CNN-RI-HMAX was 99.7% when the image contained one feature. The feature recognition accuracy decreased to 98.9% when the image contained 7 features. In Fig 9(b), in low-contrast images, SURF achieved a feature recognition accuracy of 98.4% when the image contained one feature. The feature recognition accuracy decreased to 94.2% when the image contained 7 features. The feature recognition accuracy of OMP was 97.8% when the image contained one feature. The feature recognition accuracy decreased to 94.6% when the image contained 7 features. The feature recognition accuracy of CNN-RI-HMAX in images containing one feature was 99.4%. The feature recognition accuracy decreased to 98.2% when the image contained 7 features. This indicated that this research method had better image feature extraction capabilities and provided higher quality data support for key information processing.

**Fig 10:**
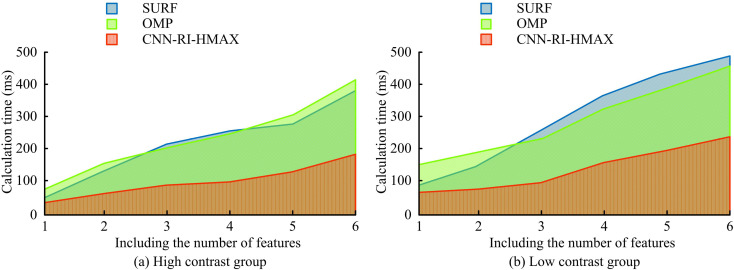
Calculation time for image key information processing.

In Fig 10, different methods increased the computational time as the image features increased when processing key image information. In Fig 10(a), in a high-contrast image, the image key information processing time of SURF when the image contained one feature was 52ms. The calculation time for processing key information in an image with 6 features was 383ms. When the image contained one feature, the OMP image key information processing time was 79ms. The calculation time for processing key information in an image with 6 features was 408ms. The processing time for image key information in CNN-RI-HMAX when the image contained one feature was 34ms. The processing time for image key information when the image contained 6 features was 183 ms. In Fig 10(b), in a low-contrast image, the image key information processing time of SURF when the image contained one feature was 94 ms. The processing time for image key information when the image contained 6 features was 492 ms. When the image contained one feature, the OMP image key information processing time was 151 ms. The processing time for key information in an image with 6 features was 458ms. The processing time for image key information using CNN-RI-HMAX when an image contained one feature was 72 ms. The processing time for image key information when the image contained 6 features was 237 ms. This indicated that the research method had faster information processing and computational efficiency.

### 4.2 Application analysis of optimized RI-HMAX image key information processing technology

**Fig 11:**
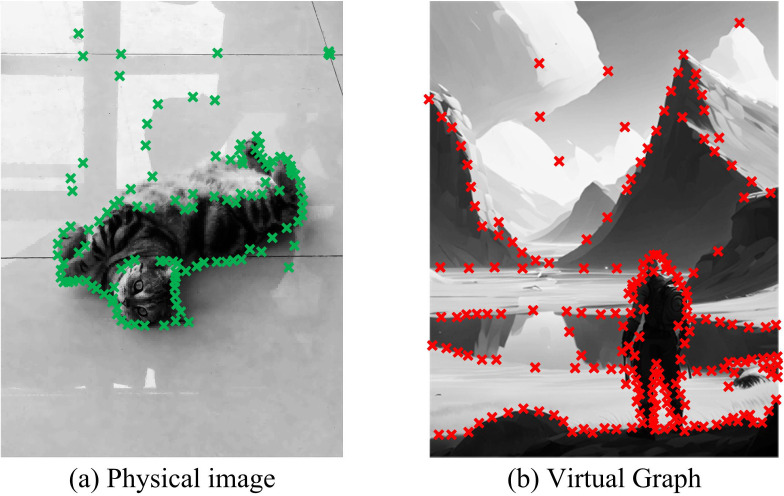
Analysis of corner extraction results.

The effectiveness of the designed image key information processing technology in practical applications was analyzed. This study extracted a physical image and a virtual image generated by artificial intelligence for key information processing. Corner extraction was performed on both physical and virtual images using CNN-RI-HMAX in Fig 11.

In Fig 11, CNN-RI-HMAX successfully completed corner extraction for both the physical and virtual images. In Fig 11(a), in the physical image, the extracted corner points included the contour of the object in the image and the obvious color difference boundary positions in the background. The corners where the contour color of the object was close to the background color were relatively sparse. The corners at the clear color difference boundary positions with no obvious significance in the background were also relatively sparse. In Fig 11(b), in the virtual image, the extracted corner points included prominent contour lines in the image. The corner distribution on the contour of the objects near the screen was relatively dense. The corner distribution on the contour of the object at the far end of the screen was relatively sparse. This indicated that the research method effectively completed the corner extraction and had the correct extraction weights.

**Fig 12:**
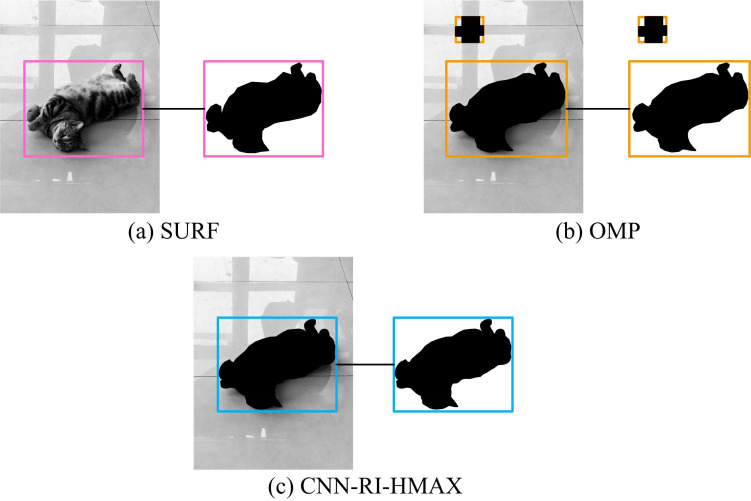
Segmentation results of physical images.

In Fig 12, there were certain differences in the segmentation results of physical images using different methods. In Fig 12(a), SURF was able to effectively extract key information objects during physical image segmentation. However, SURF lacked sufficient positioning accuracy for contours with close color differences. In Fig 12(b), OMP effectively extracted key information objects during physical image segmentation, but there was excess extraction content that needed further screening. In Fig 12(c), CNN-RI-HMAX effectively extracted key information objects and had good contour accuracy during physical image segmentation. This indicated that the research method had better image segmentation capabilities and effectively ensured the quality of key information processing.

**Fig 13:**
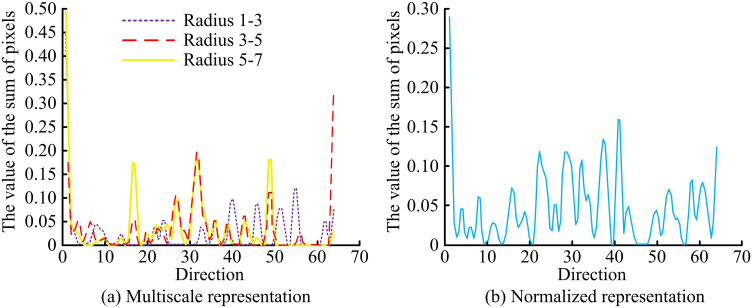
Analysis of rotational non-deformation characterization.

In Fig 13, CNN-RI-HMAX was able to extract the pixel value distribution on different radius rings of the physical image and generate normalized results. In Fig 13(a), on the 1–3 radius ring, the main peak positions of pixel sum were at positions 40, 46, 51, and 55, with peak pixel sum values above 0.07. On a 3–5 radius ring, the main peak positions of pixel sum were at positions 2, 27, 32, 49, and 64, with peak pixel sum values above 0.10. On the 5–7 radius ring, the main peak positions of pixel sum were at positions 1, 16, 32, 49, and 64, with peak pixel sum values above 0.17. In Fig 13(b), after normalization, the main peak positions of pixel sum were at positions 1, 22, 29, 32, 37, 41, and 64. The peak position of pixels reflected the real image situation, indicating that the research method was effective in extracting the representation of rotated images.

**Fig 14:**
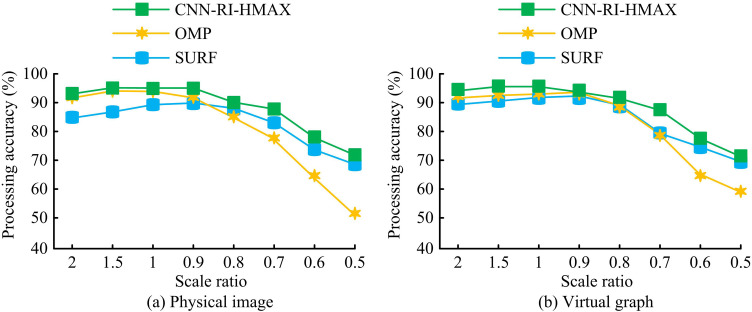
Scale ratio change test.

In Fig 14, the accuracy of image processing varied significantly with the change of image scale using different methods. In Fig 14(a), in the physical image, SURF achieved the highest image processing accuracy at a scale ratio of 0.9, which was 89.7%. The image processing accuracy of OMP reached its highest value at a scale ratio of 1.0, reaching 93.7%. The image processing accuracy of CNN-RI-HMAX reached its highest value at a scale ratio of 1.0, reaching 95.3%. In Fig 14(b), in the virtual image, SURF achieved the highest image processing accuracy at a scale ratio of 0.9, reaching 92.4%. The image processing accuracy of OMP reached its highest value at a scale ratio of 0.9, reaching 93.3%. The image processing accuracy of CNN-RI-HMAX reached its highest value at a scale ratio of 1.0, reaching 95.7%. The effectiveness of the rotational invariant module was verified and the applicability and performance of the CNN model in rotating and scaling scenarios were improved. This indicated that the research method had better accuracy in image processing. To provide further confirmation of the superiority of the research method, a comprehensive comparison was made between the research method and other existing methods. The computational efficiency of the algorithm was represented by the Processing Time index, as shown in Table 2.

**Table 2:** Comprehensive comparison of existing methods.

Method	CNN-RI-HMAX	Traditional CNNs	Average pooling	Spatial pyramid pooling
Processing time (ms)	301	312	275	254
Feature recognition accuracy (%)	98.9	92.4	94.6	95.3
Rotational invariance test accuracy (%)	96.8	87.5	89.2	91.8
Scale adaptability test accuracy (%)	95.3	84.7	87.4	89.5
Training convergence iterations	19	43	37	34
High contrast accuracy (%)	99.7	95.6	96.1	97.8
Low contrast accuracy (%)	98.2	91.3	93.5	94.2

As shown in Table 2, the average processing time of CNN-RI-HMAX was 301ms, which was lower than the 312ms of Traditional CNNs. Compared with Average Pooling and Spatial Pyramid Pooling, the increase remained within 20%. The research method did not have a significant impact on the computational efficiency of the method even after increasing the complexity of the model. CNN-RI-HMAX achieved a feature recognition accuracy of 98.9%, which was 6.5% higher than traditional CNN, 4.3% higher than average pooling, and 3.6% higher than spatial pyramid pooling, demonstrating higher recognition ability. The findings indicated that the proposed approach exhibited superior performance in terms of recognition accuracy and adaptability when compared with conventional CNNs and other pooling mechanisms, without substantial compromise to computational efficiency. This outcome serves to substantiate the efficacy of the proposed methodology.

## 5 Conclusion

An important image information processing technique combining CNN and RI-HMAX was developed to improve the data quality of image processing. Gabor filters were used to filter and compute the input image. The feature extraction content of Gabor was refined by local contrast significance detection. After Taylor expansion and return, the corner values were transformed into matrix expressions. The features were extracted synchronously using CNN, and the diagonal points were analyzed to determine the dominant direction in the image. The similarity results were mapped using a Gaussian kernel. When the research method was trained on high-contrast images, the loss value dropped to a position close to 0 and remained stable after 19 iterations. In the feature recognition accuracy test, this research method achieved a feature recognition accuracy of 98.2% in low-contrast images with 7 features, which was higher than other methods. When performing physical image segmentation, this research method extracted objects with good contour accuracy. When performing rotational deformation characterization analysis with this research method, 14 peak positions on 3 radius rings were extracted and normalized to obtain 7 peak positions. The image processing accuracy of this research method was 95.3% when the image scale ratio in the physical image was 1.0. This indicated that this research method had a better processing speed and accurately performed important image information processing tasks. However, this study only analyzed static images and did not consider the complex motion process of image content in dynamic images. In the future, the experimental scope will be expanded, the experimental results will be enriched, and the method will be optimized. Meanwhile, due to the rotation invariance configuration of the research, it possesses augmented potential for application in tasks necessitating the processing of multi-angle and complex scenes. For example, targets in remote sensing images often appear in different directions due to changes in the satellite’s shooting angle. In unmanned driving systems, the difficulty of detecting traffic signs and road environments captured by cameras can increase due to changes in angles. The use of research methods can provide technical support in these areas and promote technological progress in areas such as remote sensing image analysis, autonomous driving, and automatic navigation.

## Supporting information

S1 File Minimal data set. (DOC)
