# Supplementary material for: Image key information processing using convolutional neural network and rotational invariant-hierarchical max pooling algorithm
Source: PLoS One. 2025 May 27;20(5):e0324504. doi: 10.1371/journal.pone.0324504 (PMC12111312; doi:10.1371/journal.pone.0324504)
Supplement: S1 File — (DOC) [file pone.0324504.s001.doc]

**Data in Figure 8**

| Contrast Group | Method | Initial Loss Value | Iterations to Stability | Final Stable Loss Value |
| --- | --- | --- | --- | --- |
| High Contrast | SURF | 1.24 | 47 | close to 0 |
|  | OMP | 1.32 | 31 | close to 0 |
|  | CNN-RI-HMAX | 0.83 | 19 | close to 0 |
| Low Contrast | SURF | 1.22 | 62 | close to 0 |
|  | OMP | 0.68 | 51 | close to 0 |
|  | CNN-RI-HMAX | 0.9 | 32 | close to 0 |

**Data in Figure 9**

| Method | Including the number of features | Recognition accuracy (%) | |
| --- | --- | --- | --- |
|  |  | High contrast | Low contrast |
| SURF | 1 | 99.1 | 98.4 |
|  | 3 | 97.3 | 96.2 |
|  | 5 | 95.8 | 95.3 |
|  | 7 | 94.6 | 94.2 |
| OMP | 1 | 98.7 | 97.8 |
|  | 3 | 97.9 | 96.9 |
|  | 5 | 97.2 | 95.8 |
|  | 7 | 96.8 | 94.6 |
| CNN-RI-HMAX | 1 | 99.7 | 99.4 |
|  | 3 | 99.4 | 99.1 |
|  | 5 | 99.2 | 98.7 |
|  | 7 | 98.9 | 98.2 |

**Data in Figure 10**

| Method | Including the number of features | Calculation time (ms) | |
| --- | --- | --- | --- |
|  |  | High contrast | Low contrast |
| SURF | 1 | 52 | 94 |
|  | 2 | 98 | 128 |
|  | 3 | 142 | 187 |
|  | 4 | 205 | 251 |
|  | 5 | 312 | 364 |
|  | 6 | 383 | 492 |
| OMP | 1 | 79 | 151 |
|  | 2 | 121 | 201 |
|  | 3 | 184 | 274 |
|  | 4 | 248 | 343 |
|  | 5 | 325 | 398 |
|  | 6 | 408 | 458 |
| CNN-RI-HMAX | 1 | 34 | 72 |
|  | 2 | 68 | 97 |
|  | 3 | 115 | 131 |
|  | 4 | 144 | 167 |
|  | 5 | 161 | 204 |
|  | 6 | 183 | 237 |

**Data in Figure 13(a)**

| Range of radius | Peak position | Peak pixel value |
| --- | --- | --- |
| Radius 1-3 | 40, 46, 51, 55 | > 0.07 |
| Radius 3-5 | 2, 27, 32, 49, 64 | > 0.10 |
| Radius 5-7 | 1, 16, 32, 49, 64 | > 0.17 |

**Data in Figure 13(b)**

| Peak position | The value of the sum of pixels |
| --- | --- |
| 1 | > 0.15 |
| 22 | > 0.20 |
| 29 | > 0.25 |
| 32 | > 0.30 |
| 37 | > 0.35 |
| 41 | > 0.40 |
| 64 | > 0.45 |

**Data in Figure 14**

| Method | Scale ratio | Processing accuracy (%) | |
| --- | --- | --- | --- |
|  |  | Physical image | Virtual graph |
| SURF | 0.5 | 76.3 | 78.5 |
|  | 0.6 | 81.4 | 84.2 |
|  | 0.7 | 84.9 | 88.1 |
|  | 0.8 | 87.2 | 90.3 |
|  | 0.9 | 89.7 | 92.4 |
|  | 1.0 | 88.9 | 91.8 |
| OMP | 0.5 | 80.2 | 81.6 |
|  | 0.6 | 84.7 | 86.8 |
|  | 0.7 | 88.3 | 90.7 |
|  | 0.8 | 91.5 | 92.6 |
|  | 0.9 | 93.4 | 93.3 |
|  | 1.0 | 93.7 | 92.9 |
| CNN-RI-HMAX | 0.5 | 85.1 | 87.4 |
|  | 0.6 | 89.3 | 91.2 |
|  | 0.7 | 92.1 | 93.7 |
|  | 0.8 | 94.3 | 95.0 |
|  | 0.9 | 95.0 | 95.4 |
|  | 1.0 | 95.3 | 95.7 |
